# Supplementary material for: Oxalic Acid Inhibits Feeding Behavior of the Brown Planthopper via Binding to Gustatory Receptor Gr23a
Source: Cells. 2023 Feb 28;12(5):771. doi: 10.3390/cells12050771 (PMC10001216; doi:10.3390/cells12050771)
Supplement: Supplementary file 1 [file cells-12-00771-s001.zip › cells-2196980-supplementary.pdf]

```
1 ATGCTGAAGAACAAGCGCCTGCAAAGTAACAAGAGAGGTATCGGGAGACTGCTGGATATG
1   M  L  K  N  K  R  L  Q  S  N  K  R  G  I  G  R  L  L  D  M
61 AGAAACAACAAAGCCGCCACCAAACGCGTCACCTCCCTGCCTCACATTATACATGTGCTG
21  R  N  N  K  A  A  T  K  R  V  T  S  L  P  H  I  I  H  V  L
121GATGTTAACTATAAGACAGCCTATGATTATCATGAGAAGCTGAGGCACTGTATCTACTTC
41  D  V  N  Y  K  T  A  Y  D  Y  H  E  K  L  R  H  C  I  Y  F
181GAACCTATTCAAATAGTCCTCTTCGTCTGGAGCCTGCTGGGCAGACTGCCAATCAGACGC
61  E  P  I  Q  I  V  L  F  V  W  S  L  L  G  R  L  P  I  R  R
241GACTCTACTGGAGACTATATTGTGCCCATTGTCCTCAGAGTGTACAGTTTCCTCGTTGGA
81  D  S  T  G  D  Y  I  V  P  I  V  L  R  V  Y  S  F  L  V  G
301AGTATCATTGTGGTGATTGGCTCAATCAGCCTGAGGAACCTCGTCAAGTCCCTCCTGAAC
101 S  I  I  V  V  I  G  S  I  S  L  R  N  L  V  K  S  L  L  N
361GGCGAGAGCGTGTACGAAGAGCCCACATCTGTGATTGGAGACTACAACTATACACTCTTT
121 G  E  S  V  Y  E  E  P  T  S  V  I  G  D  Y  N  Y  T  L  F
421TGGATCTGTCAGATCCTCGTGACCACTTATAACATTTCCCTGCCCTTCCTGGACTGGACT
141 W  I  C  Q  I  L  V  T  T  Y  N  I  S  L  P  F  L  D  W  T
481ATCGTGGGCGAGTTCCTCAAGTTTCTCCGCAGCTGGGCTCAGTTCCAGGAGAACTATACC
161 I  V  G  E  F  L  K  F  L  R  S  W  A  Q  F  Q  E  N  Y  T
541CTGGAGAGCCGGAAAGTGCTGAGCATCAAGAGCAAGAACAGAATACGCATCATCTTCTAT
181 L  E  S  R  K  V  L  S  I  K  S  K  N  R  I  R  I  I  F  Y
601ATGACTCTGCTGGTGAACATGACCACCGTCGCCTACCTGAACAATTTCGTGATGAAGAAC
201 M  T  L  L  V  N  M  T  T  V  A  Y  L  N  N  F  V  M  K  N
661GTGGATGTGTTTACCAAGATCAGTACCCTCACTGCAGTCGTGGAACTCGAATCCCGCACT
221 V  D  V  F  T  K  I  S  T  L  T  A  V  V  E  L  E  S  R  T
721GCTTTCTTCTGGCATACCTGTGCTGTCATCAGCGATTGCAGCGAACAACTGTCTATCACC
241 A  F  F  W  H  T  C  A  V  I  S  D  C  S  E  Q  L  S  I  T
781TTTTGCAAGAAAACCCTGGAGTCCAGAGTGAGCAGAGGCGCACAGACACTCGGAGATTAC
261 F  C  K  K  T  L  E  S  R  V  S  R  G  A  Q  T  L  G  D  Y
841AGGCGCCTCTGGCTGCAGCTGACCGACATTACTACTAGTTTCAGCGATCTCATGAGCTAC
281 R  R  L  W  L  Q  L  T  D  I  T  T  S  F  S  D  L  M  S  Y
901AAGATGACCCATTCAATCTTTGTTAATGTGATTTTCGTTATAGTGACTAGTTATCATCTG
301 K  M  T  H  S  I  F  V  N  V  I  F  V  I  V  T  S  Y  H  L
961GCCCTGTCAGCACTGAGCCGCGGAGTTCGCGGCAGTGCTGGAGACATATACCTCTACTTC
321 A  L  S  A  L  S  R  G  V  R  G  S  A  G  D  I  Y  L  Y  F
1021TTCTGTTCCCTCTACACAGCAGCCCACGTGATGCTGGCCTGTGAATCCGGCCACAGACTC
341 F  C  S  L  Y  T  A  A  H  V  M  L  A  C  E  S  G  H  R  L
1081GCTCAGAGTCTCGGGAAGAAATTTGTTGATCGGGCCACCAGGCGGAGACTCCAACACCTG
361 A  Q  S  L  G  K  K  F  V  D  R  A  T  R  R  R  L  Q  H  L
1141TCATTCGCAGAGCGGAAAGAGTTTTCTGTGTTTATGGGCATCGTGCGCAGGAGGTCACCT
381 S  F  A  E  R  K  E  F  S  V  F  M  G  I  V  R  R  R  S  P
1201TCAGTCATCCTCAATGGTTTTCTCACCGTCGATCGCAGTCTGATCGTGCTCTTTGTGAGT
401 S  V  I  L  N  G  F  L  T  V  D  R  S  L  I  V  L  F  V  S
1261GGATGTCTCACATACCTGATCATTCTCGTGCAATTCAAAGTGGGCATGAGCGAAACTCAT
421 G  C  L  T  Y  L  I  I  L  V  Q  F  K  V  G  M  S  E  T  H
1321 AGAGTGCAGAATAGCAATATCTCACTCAGCAGG
441 R  V  Q  N  S  N  I  S  L  S  R
```

Figure S1. The nucleotide sequences of human codon-optimized *NlGr23a* genes.

```
CATAATAAAATATGTTTTCATGTATGAAATAAAATAATAGATCGAACTAGCAAAAGTCTTGATATATATG CTG AAA AAT AAA AGG TTG
                                                                       M   L   K   N   K   R   L
CAA TCG AAT AAA AGA GGT ATT GGA AGA TTA TTA GAC ATG AGA AAT AAT AAG GCG GCG ACT AAG CGA GTG ACG
 Q   S   N   K   R   G   I   G   R   L   L   D   M   R   N   N   K   A   A   T   K   R   V   T
TCA CTT CCT CAT ATT ATT CAC GTA CTT GAT GTC AAT TAC AAA ACT GCT TAT GAC TAT CAT GAA AAA TTG CGA
 S   L   P   H   I   I   H   V   L   D   V   N   Y   K   T   A   Y   D   Y   H   E   K   L   R
CAT TGC ATC TAT TTT GAG CCG ATT CAA ATT GTA TTA TTT GTG TGG TCT TTA TTA GGC CGG TTA CCT ATC AGG
 H   C   I   Y   F   E   P   I   Q   I   V   L   F   V   W   S   L   L   G   R   L   P   I   R     TM1
CGA GAT TCA ACA GGA GAT TAC ATC GTA CCA ATC GTC CTC CGG GTC TAT TCT TTC CTA GTT GGC TCT ATA ATA
 R   D   S   T   G   D   Y   I   V   P   I   V   L   R   V   Y   S   F   L   V   G   S   I   I     TM2
GTT GTT ATT GGC AGT ATA TCT CTT CGA AAT CTA GTT AAA AGT TTG CTG AAT GGC GAA TCT GTA TAT GAA GAG
 V   V   I   G   S   I   S   L   R   N   L   V   K   S   L   L   N   G   E   S   V   Y   E   E
CCC ACG TCA GTG ATA GGC GAT TAC AAC TAC ACA CTG TTC TGG ATA TGT CAG ATA CTG GTA ACA ACT TAT AAC
 P   T   S   V   I   G   D   Y   N   Y   T   L   F   W   I   C   Q   I   L   V   T   T   Y   N     TM3
ATC AGT TTA CCA TTT TTG GAC TGG ACT ATA GTA GGC GAA TTT CTC AAA TTC CTC CGC AGT TGG GCC CAA TTC
 I   S   L   P   F   L   D   W   T   I   V   G   E   F   L   K   F   L   R   S   W   A   Q   F
CAA GAG AAT TAC ACA CTG GAA AGT AGA AAA GTA CTG TCT ATA AAA TCC AAA AAT AGA ATC AGA ATA ATA TTC
 Q   E   N   Y   T   L   E   S   R   K   V   L   S   I   K   S   K   N   R   I   R   I   I   F     TM4
TAC ATG ACT CTG CTG GTA AAT ATG ACA ACG GTT GCA TAT CTC AAC AAT TTC GTG ATG AAA AAT GTC GAC GTT
 Y   M   T   L   L   V   N   M   T   T   V   A   Y   L   N   N   F   V   M   K   N   V   D   V
TTT ACT AAA ATA TCA ACT TTA ACT GCA GTT GTT GAG CTG GAG AGT CGC ACT GCT TTT TTC TGG CAT ACT TGT
 F   T   K   I   S   T   L   T   A   V   V   E   L   E   S   R   T   A   F   F   W   H   T   C
GCA GTT ATT TCC GAT TGT TCG GAG CAG CTC TCA ATC ACG TTT TGT AAG AAG ACA CTG GAG TCC CGA GTG TCA
 A   V   I   S   D   C   S   E   Q   L   S   I   T   F   C   K   K   T   L   E   S   R   V   S
AGA GGC GCA CAA ACA TTG GGT GAC TAC AGA AGG TTG TGG CTG CAG TTG ACG GAC ATC ACA ACA AGT TTC AGC
 R   G   A   Q   T   L   G   D   Y   R   R   L   W   L   Q   L   T   D   I   T   T   S   F   S
GAC TTG ATG AGC TAC AAA ATG ACT CAC TCA ATA TTC GTG AAC GTT ATT TTC GTG ATA GTA ACA AGC TAT CAC
 D   L   M   S   Y   K   M   T   H   S   I   F   V   N   V   I   F   V   I   V   T   S   Y   H     TM5
CTG GCT CTG TCA GCT CTA TCG CGA GGT GTG AGG GGC AGT GCA GGC GAC ATC TAT CTC TAT TTT TTC TGC AGT
 L   A   L   S   A   L   S   R   G   V   R   G   S   A   G   D   I   Y   L   Y   F   F   C   S     TM6
CTG TAC ACA GCG GCG CAC GTG ATG CTT GCG TGC GAG AGT GGA CAC AGA CTG GCT CAA AGT CTA GGC AAG AAG
 L   Y   T   A   A   H   V   M   L   A   C   E   S   G   H   R   L   A   Q   S   L   G   K   K
TTT GTG GAC AGA GCA ACT CGT CGC AGG TTG CAG CAT CTC TCG TTT GCC GAG CGG AAA GAG TTC AGT GTG TTC
 F   V   D   R   A   T   R   R   R   L   Q   H   L   S   F   A   E   R   K   E   F   S   V   F
ATG GGC ATT GTG AGA CGT CGG TCG CCG AGT GTT ATA CTC AAC GGT TTC CTG ACG GTC GAC CGC AGT CTG ATC
 M   G   I   V   R   R   R   S   P   S   V   I   L   N   G   F   L   T   V   D   R   S   L   I
GTT CTT TTT GTC TCC GGG TGT CTC ACT TAT CTC ATC ATT CTC GTA CAG TTC AAA GTG GGG ATG TCG GAG ACC
 V   L   F   V   S   G   C   L   T   Y   L   I   I   L   V   Q   F   K   V   G   M   S   E   T     TM7
CAT AGG GTA CAA AAC AGC AAT ATC AGT CTC TCG AGG TAGTAGAATTTGTATTTTAGAAAATAATTTGAGCATTGTGTATCAGA
 H   R   V   Q   N   S   N   I   S   L   S   R   *
ATTCTTATTTGGTATTCTGGAATGTAATTAGTTGTATACTAAGTCTATCATACTACTGTACATCATAAATTGCTACCATTGCTGATCCAAGTTGA
TGGTTTAAAAGTAAGACAATTTCAATGTAAGACAATGTTTTTTATTCTCTATTATTGTTATACTTTCAAGTGTATTATTGGTTGTGGCGTTATTG
AGGATAATGAGCTTCATTTGCTACAATAAAAATTTCATTCAACTTATACACTATTGCTTTTCATAGTCCAATCGTCCA
```

Figure S2. The gene structure of *NlGr23a*. Nucleotide and deduced amino acid sequences of *NlGr23a* cDNA. The predicted seven transmembrane domains (TMs) are shaded in grey. "*" represents the terminal codon.

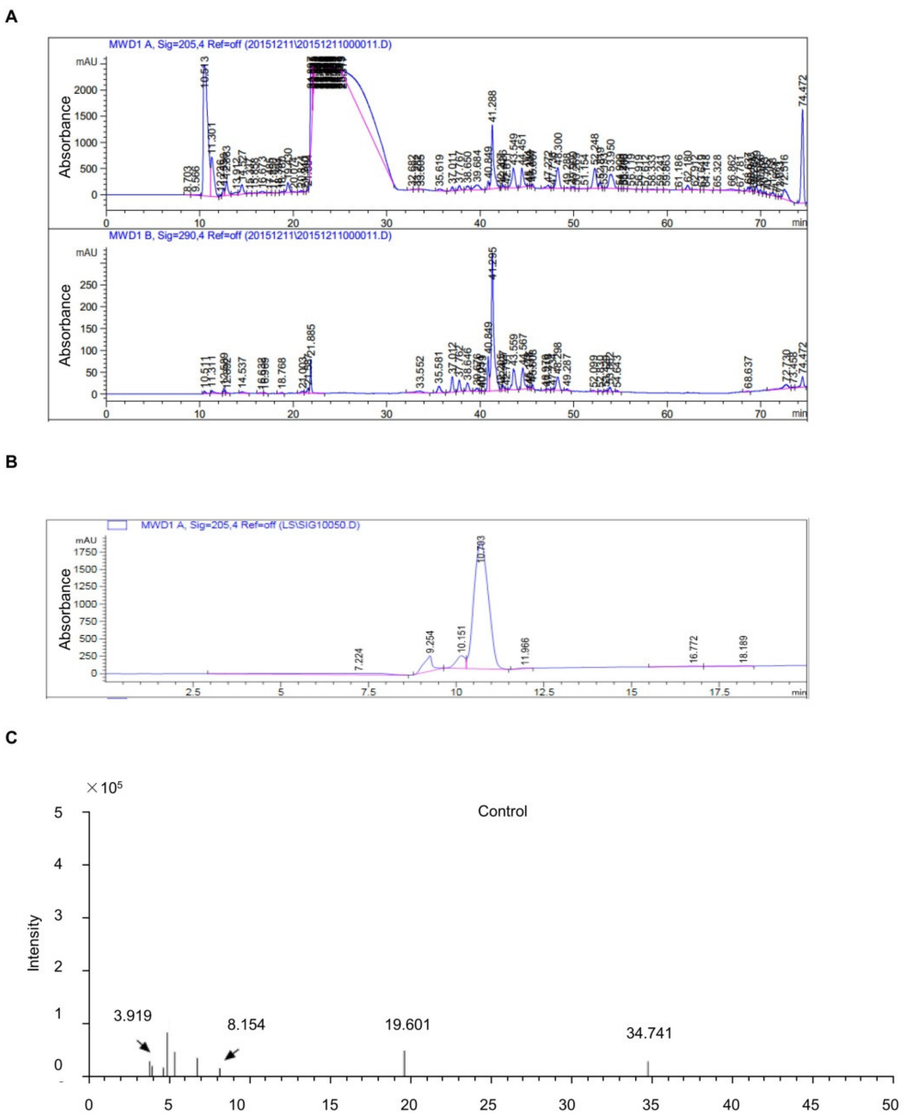

Figure S3. Isolation and identification of rice crude extracts. (**A**) HPLC chromatogram of the ethyl acetate fraction. The UV detection wavelengths were set at 205 nm (top) and 290 nm (bottom). (**B**) HPLC chromatogram of bioassay-positive fraction from round 2 of ligand screening. The UV detection wavelength was set at 205 nm. (**C**) GC-MS chromatogram of the control solution. Arrowheads indicate the retention time of potential ligands identified in the bioassay-positive fraction. In the control solution, the intensities of theses potential ligands examined by GC-MS chromatogram were low.

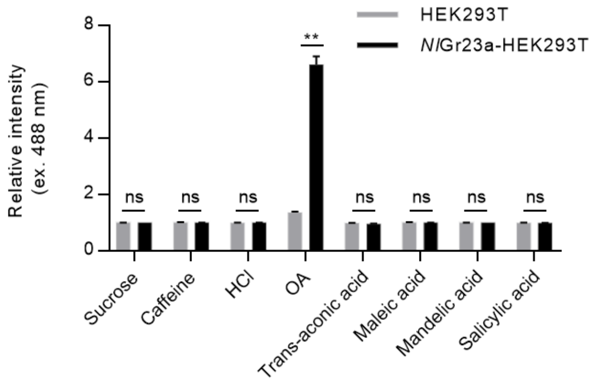

Figure S4. Responses of the *Nl*Gr23a-HEK293T cells to different phytochemicals. Ca^{2+} response of the *Nl*Gr23a-expressing HEK293T cells ($n \geq 49$) was stimulated with indicated tastants (each at 10 mM) and indicated by Fluo4 intensity which was normalized to the baseline before stimulation. Bars represent means ± SEM. Mann-Whitney non parametric test; ns means no statistical difference (p >0.05); **p <0.01.

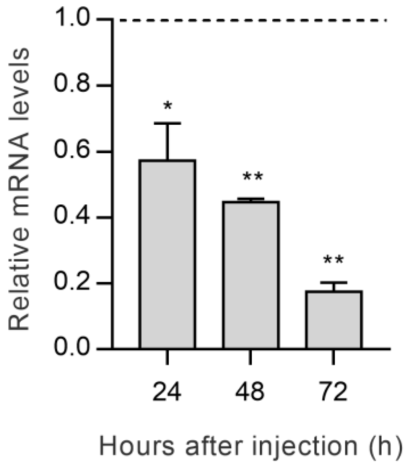

Figure S5. *NlGr23a* expression levels in BPHs after RNAi. The messenger RNA (mRNA) levels of *NlGr23a* in BPHs 24, 48 and 72 h after injection of *NlGr23a* dsRNA. Injection of ds*GFP* was used as the control. All mRNA levels were normalized relative to the *β*-actin mRNA levels. The *NlGr23a* mRNA level in controls was set as 1. Bars represent means + SEM. Mann-Whitney non parametric test; *p < 0.05; **p < 0.01 (n = 3).

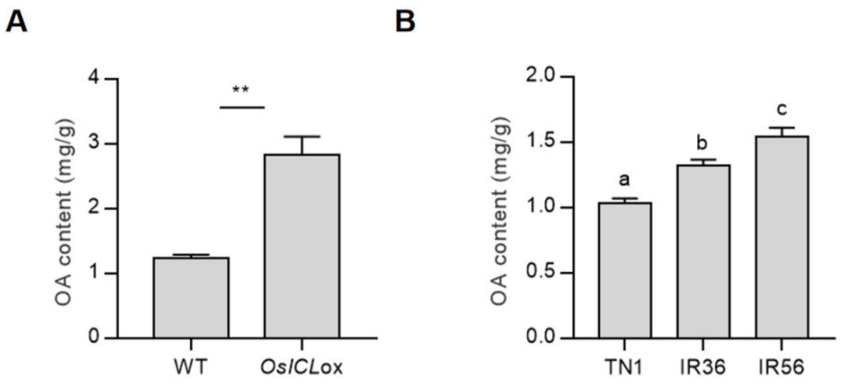

Figure S6. OA content in different rice plants. (**A**) OA content in WT and *OsICL*ox plants. Bars represent means ± SEM. Mann-Whitney non parametric test; **$p < 0.01$ ($n = 3$). (**B**) OA content in different rice varieties. Bars represent means ± SEM. Different letters (a to c) above bars represent significantly different groups. Multiple samples were analysed using ANOVA at $p < 0.05$ followed by Duncan's multiple range test ($n = 4$).

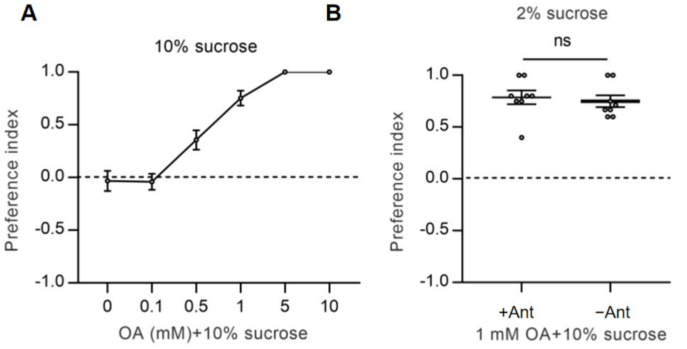

Figure S7. OA induced feeding avoidance. (**A**) Dose-responsive food choice assay for OA using an equal concentration of sucrose. BPHs were given the choice between 10% sucrose solution and 10% sucrose mixed with different concentrations of OA. Bars represent means ± SEM ($n = 8$). (**B**) Role of olfaction in OA avoidance. Food choice assay was performed with insects removed antennae (−Ant) or not (+Ant). Bars represent means ± SEM. Mann-Whitney non parametric test; ns, $p > 0.05$ ($n = 8$).

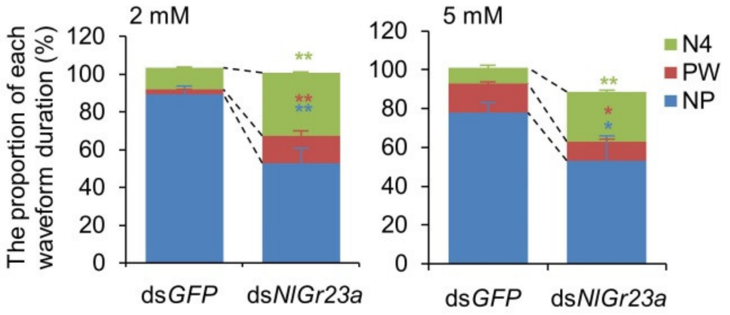

Figure S8. The duration of each waveform as a proportion of observation time produced by ds*NlGr23a*- and ds*GFP*- treated BPHs on LDS containing artificial diets with 2 mM or 5 mM OA. Stacked bar graphs represent means + SEM. Mann-Whitney non parametric test; *$p < 0.05$; **$p < 0.01$ ($n = 5$). There are three typical waveforms, including non-penetration (NP), pathway (PW), and ingestion (N4).

Table S1. Primers used in this study.

Primer Name	Sequence (5'–3')
NlGr23a-ORF-F	ATGCTGAAAAATAAAAGGTTGCAATC
NlGr23a-ORF-R	CTCGAGAGACTGATATTGCTGTT
NlGr23a-HindIII-F	CCCAAGCTTATGCTGAAAAATAAAAGGTTGCAATC
NlGr23a-EcoRI-R	CGGAATTCCCTCGAGAGACTGATATTGCTGTT
NlGr23a-dsRNA-F	TTACCATTTTTGGACTGGACTA
NlGr23a-dsRNA-R	ACTGCAGTTAAAGTTGATATT
T7-*NlGr23a*-dsRNA-F	GGATCCTAATACGACTCACTATAGGATTACCATTTTT-GGACTGGACTA
T7-*NlGr23a*-dsRNA-R	GGATCCTAATACGACTCACTATAGGACTGCAGTTAAAGTT-GATATT
ds*GFP*-F	AAGGGCGAGGAGCTGTTCACCG
ds*GFP*-R	CAGCAGGACCATGTGATCGCGC
T7-ds*GFP*-F	GGATCCTAATACGACTCACTATAGGAAGGGCGAG-GAGCTGTTCACCG
T7-ds*GFP*-R	GGATCCTAATACGACTCACTATAGGACAGCAGGAC-CATGTGATCGCGC
Q-*β-actin*-F	TGCGTGACATCAAGGAGAAGC
Q-*β-actin*-R	CCATACCCAAGAAGGAAGGCT
Q-*NlGr23a*-F	TAGGCAAGAAGTTTGTGGACAG
Q-*NlGr23a*-R	ATGAGATAAGTGAGACACCCGG

Table S2. Identification of differential peaks by GC-MS.

**Retention Time (min)**		**Intensity**		**Name**
Control	Sample	Control	Sample	
3.919	3.909	22228	100977	Oxalic acid
8.154	8.144	18117	445691	Glycerol
19.601	19.581	51353	62489	Phthalic acid
34.741	34.691	31094	48765	Trisiloxane
